# H_2_S remodels mitochondrial ultrastructure and destabilizes respiratory supercomplexes

**DOI:** 10.1016/j.jbc.2025.108433

**Published:** 2025-03-20

**Authors:** David A. Hanna, Brandon Chen, Yatrik M. Shah, Oleh Khalimonchuk, Brian Cunniff, Ruma Banerjee

**Affiliations:** 1 Department of Biological Chemistry, Michigan Medicine, Ann Arbor, Michigan, USA; 2 Department of Molecular and Integrative Physiology, Michigan Medicine, Ann Arbor, Michigan, USA; 3 Department of Cellular and Molecular Biology Program, Michigan Medicine, Ann Arbor, Michigan, USA; 4 Department of internal Medicine (Division of Gastroenterology), Michigan Medicine, Ann Arbor, Michigan, USA; 5 Department of Biochemistry, University of Nebraska-Lincoln, Lincoln, Nebraska, USA; 6 Department of Pathology and Laboratory Medicine, University of Vermont Larner College of Medicine, Burlington, Vermont, USA

**Keywords:** cristae, electron transport chain, hydrogen sulfide, mitochondrial dynamics, respirasome

## Abstract

Mitochondrial form and function are intimately interconnected, responding to cellular stresses and changes in energy demand. Hydrogen sulfide, a product of amino acid metabolism, has dual roles as an electron transport chain substrate and complex IV (CIV) inhibitor, leading to a reductive shift, which has pleiotropic metabolic consequences. Luminal sulfide concentration in the colon is high due to microbial activity, and in this study, we demonstrate that chronic sulfide exposure of colonocyte-derived cells leads to lower Mic60 and Mic19 expression that is correlated with a profound loss of cristae and lower mitochondrial networking. Sulfide-induced depolarization of the inner mitochondrial membrane activates Oma1-dependent cleavage of Opa1 and is associated with a profound loss of CI and CIV activities associated with respirasomes. Our study reveals a potential role for sulfide as an endogenous modulator of mitochondrial dynamics and suggests that this regulation is corrupted in hereditary or acquired diseases associated with elevated sulfide.

Mitochondria are central hubs of energy metabolism that support oxidative phosphorylation *via* proton-coupled electron transfer through complexes in the electron transport chain (ETC), which are located in cristae. The latter are dynamic sac-like compartments formed by the inner mitochondrial membrane whose shape changes in response to physiological conditions (1). Arrays of dimeric ATP synthase induce curvature at cristae ridges while the proton-pumping complexes I, III, and IV (referred to as CI, CIII and CIV) but not CII are organized in supercomplexes or respirasomes of varying stoichiometry on cristae flanks (2). Cristae dynamics influence respiratory supercomplex stability and function and cristae disruption is associated with supercomplex destabilization (3). While the physiological relevance of supercomplexes remains elusive, roles such as protection against excessive reactive oxygen species (ROS) (4) formation, tuning electron flux for optimal substrate utilization (5), and boosting efficiency *via* proximity effects (6, 7), have been reported.

Cristae junction architecture is maintained by MICOS (mitochondrial contact site and cristae organizing system), the GTPase Opa1 (optic atrophy-1), and other proteins (1). The constitutive YME1L and the stress-responsive Oma1 proteases cleave Opa1 into various short isoforms. A range of stimuli, including oxidants, ATP depletion, and dissipation of the transmembrane potential, activate Oma1 influencing mitochondrial networking and connectivity (8, 9) and highlighting the interconnection between cristae morphology and organelle dynamics (10). Mic19, a key stabilizing component of MICOS, and the oligomeric stability of the metalloprotease Oma1 are both sensitive to redox changes (11, 12). Regulation of mitochondrial form and function by the transmembrane potential and by changes in the ambient redox state is a strategy for responding to changes in energy demand.

Hydrogen sulfide (H_2_S) is a potential candidate for modulating mitochondrial form and function, *via* its dual ability to interact directly with the ETC and its role as a signaling molecule (13, 14, 15, 16). H_2_S is synthesized in the cytosol and mitochondria (17, 18), and readily permeates membranes (19). ER stress, amino acid restriction, and hypoxia increase H_2_S synthesis (20, 21, 22, 23). At low concentrations, H_2_S serves as an ETC substrate, reducing CoQ, as it is itself oxidized to glutathione persulfide by sulfide quinone oxidoreductase (SQOR) (24) (Fig. 1*A*), and potentially, by reducing cytochrome c (25). At higher concentrations, H_2_S inhibits complex IV, leading to respiratory inhibition that is long-lived (26), which in turn, induces a reductive shift in redox cofactors like NAD^+^ and CoQ (27, 28) and increases aerobic glycolysis (29) and lipid biogenesis (30). Inhibition of forward electron transfer by sulfide is predicted to ripple out into the intermembrane space, impairing the Mia40-dependent oxidative protein folding pathway, which uses cytochrome c as an electron acceptor and is required for Mic19 mitochondrial localization (11, 31) (Fig. 1*A*). Alternatively, H_2_S could affect protein stability and function *via* persulfidation, an oxidative cysteine modification, that results from the reaction of sulfide with an oxidized cysteine, *e.g.*, a sulfenic acid or disulfide (16, 32).

**Figure 1:**
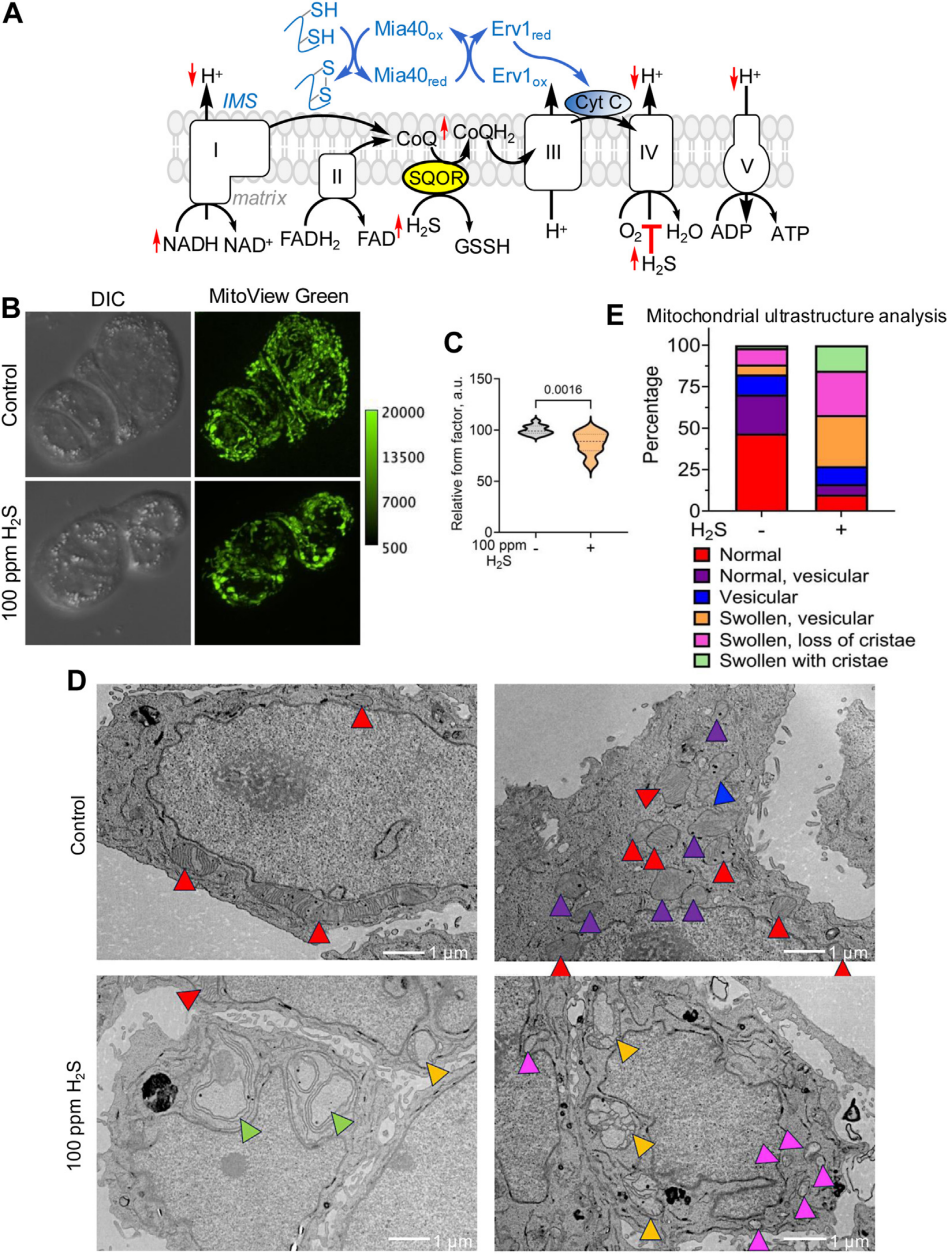
**H_2_S alters mitochondrial networking and ultrastructure.***A*, scheme illustrating the interactions of H_2_S with the ETC that lead to a reductive shift in cofactor pools and is predicted to do the same in the intermembrane space (IMS) impairing the Mia40-dependent pathway for oxidative protein folding. The *red* arrows indicate the anticipated direction of change in response to chronic H_2_S exposure. *B*, representative microscopy images of HT-29 cells stained with MitoView *Green*, reveals altered mitochondrial morphology and disrupted mitochondrial networking in response to H_2_S (100 ppm, 24 h). The calibration bar shows that the pixel intensity is within detector’s range, *i.e.*, 0 to 65,535. *C*, mitochondrial networks were estimated from the relative form factor of mitochondria in each control (n = 11) and sulfide (n = 13) image acquired from two independent experiments conducted in duplicate. *D*, representative TEM images of HT-29 cells cultured ± H_2_S (100 ppm, 24 h) show changes in mitochondrial morphology that were characterized in 28 control and 46 H_2_S treated cells from TEM sections made from biological duplicates (scale bar: 1 μm). *E*, the distribution of mitochondrial subtypes was expressed as a percentage of total mitochondria (n = 353 and 427 mitochondria without and with H_2_S treatment), using the same color scheme as in *D*.

Steady-state tissue levels of H_2_S are low and vary between 10 and 80 nM (33, 34), but its concentration is significantly higher in the colon lumen (0.2–2.4 mM) (35, 36), where it is influenced by diet and microbiota. Hereditary defects in the sulfide oxidation pathway enzymes lead to pathological elevation in sulfide and are associated with mitochondrial encephalomyopathies (37, 38). Exposure to a sublethal dose of sulfide (1000 ppm, 30 min) causes extensive mitochondrial damage in the murine brain that is characterized by swelling and fragmentation, loss of cristae, and an increase in vesicular ultrastructure (39). The potential for physiologically relevant concentrations of sulfide to modulate mitochondrial form and function is, however, unknown.

In the current study, we examined the effects of chronic exposure to low sulfide on mitochondrial ultrastructure and networking in several cell types with a focus on colonocytes. For this, we utilized our recently described sulfide incubator to administer a constant supply of H_2_S gas (100 ppm) in 5% CO_2_-containing air to cells in culture, which leads to the stable accumulation of 20 μM dissolved sulfide in the culture medium (40). Under these conditions, cells exhibit S-phase cell cycle arrest and diminished mitochondrial networks, which were reversed upon removal of H_2_S from the atmosphere, signaling an adaptive response. H_2_S elicited membrane depolarization that was correlated with an increase in Oma1, a decrease in long Opa1 isoforms, and a corresponding increase in short Opa1 isoforms. A closer examination revealed cristae loss and decreased CI and CIV activity associated with CI + CIII_2_ + CIV_n_ and CIII_2_ + CIV, which was correlated with a loss of the scaffold protein SCAF1 (or COX7A2L) from these supercomplexes. Finally, sulfide decreased levels of the ETC subunits GRIM19, NDUFB8, SCAF1, and COX7A2 and the CIV core subunits, MT-CO1 and MT-CO2. By providing a mechanistic framework linking sulfide to alterations in cristae dynamics, ETC stability, and function, our study offers insights into how physiological fluctuations and pathological elevation in H_2_S might impact mitochondrial architecture and behavior.

## Results

### H_2_S remodels mitochondrial dynamics and cristae organization

Live cell imaging revealed profound effects of H_2_S on mitochondrial networking in HT-29 cells as revealed by form factor analysis, which is a measure of mitochondrial length and the degree of branching (41) (Fig. 1, *B* and *C*). This effect was reversed within 48 h of sulfide removal (Fig. S1*A*). An H_2_S-induced decrease in mitochondrial networks was also seen in HEK293 (human embryonic kidney), HT1080 (human fibrosarcoma), and SW480 (human colon carcinoma) cells, which displayed more punctate mitochondria (Figs. S2–S4). While these cell lines exhibited some variations in mitochondrial morphology in response to H_2_S, they exhibited an overall decrease in mitochondrial networking. For the remainder of the study, we focused on HT-29 cells for characterizing changes in mitochondrial ultrastructure in response to H_2_S.

Since mitochondrial dynamics are responsive to environmental and cellular stresses, and are central to functional regulation (42), we examined how sulfide exposure affects mitochondrial ultrastructure. Transmission electron microscopy (TEM) revealed mitochondrial swelling and cristae loss in sulfide-grown cells (Figs. 1, *D* and *E*, S5). In contrast to untreated controls, in which most mitochondria (∼70%) exhibited normal or normal-vesicular morphology, >70% of sulfide-grown cells had swollen, swollen-vesicular mitochondria with partial or complete loss of cristae (Fig. 1*E*). In fact, only ∼10% of sulfide-grown cells retained normal morphology and >25% showed complete loss of cristae. Morphological changes were observed in most mitochondria irrespective of their size, and H_2_S-grown cells appeared to contain both smaller as well as much larger mitochondria relative to control cells.

Chronic H_2_S exposure (100 ppm, 24 h) diminished basal respiration in HT-29 cells by ∼90% (Fig. S6), which as reported previously, is associated with increased aerobic glycolysis (40). Culture in the sulfide chamber also prevented subsequent H_2_S-stimulated oxygen consumption, which serves as a proxy for mitochondrial dysfunction, since sulfide oxidation requires a functional ETC. These effects were reversed within 48 h of the cells being moved to a growth chamber lacking sulfide as also observed at the level of mitochondrial morphology (Fig. S1*A*). We have previously shown that under these conditions, HT-29 cells exhibit decreased proliferation albeit, without a significant effect on cell viability (40). Cell cycle analysis revealed that sulfide (100 ppm, 24 h) increased the proportion of cells in the S-phase, which slowly redistributed over the next 48 h following removal of the sulfide from the growth chamber (Fig. S7). The G1-S transition is characterized by hyperfused mitochondria that are electrically continuous and have a higher ATP-generating potential than at any other phase of the cell cycle (43). A combination of fragmented and hyperfused mitochondria was seen in sulfide-grown cells reflecting S-phase enrichment.

### H_2_S disrupts inner membrane organization by destabilizing MICOS

**Figure 2:**
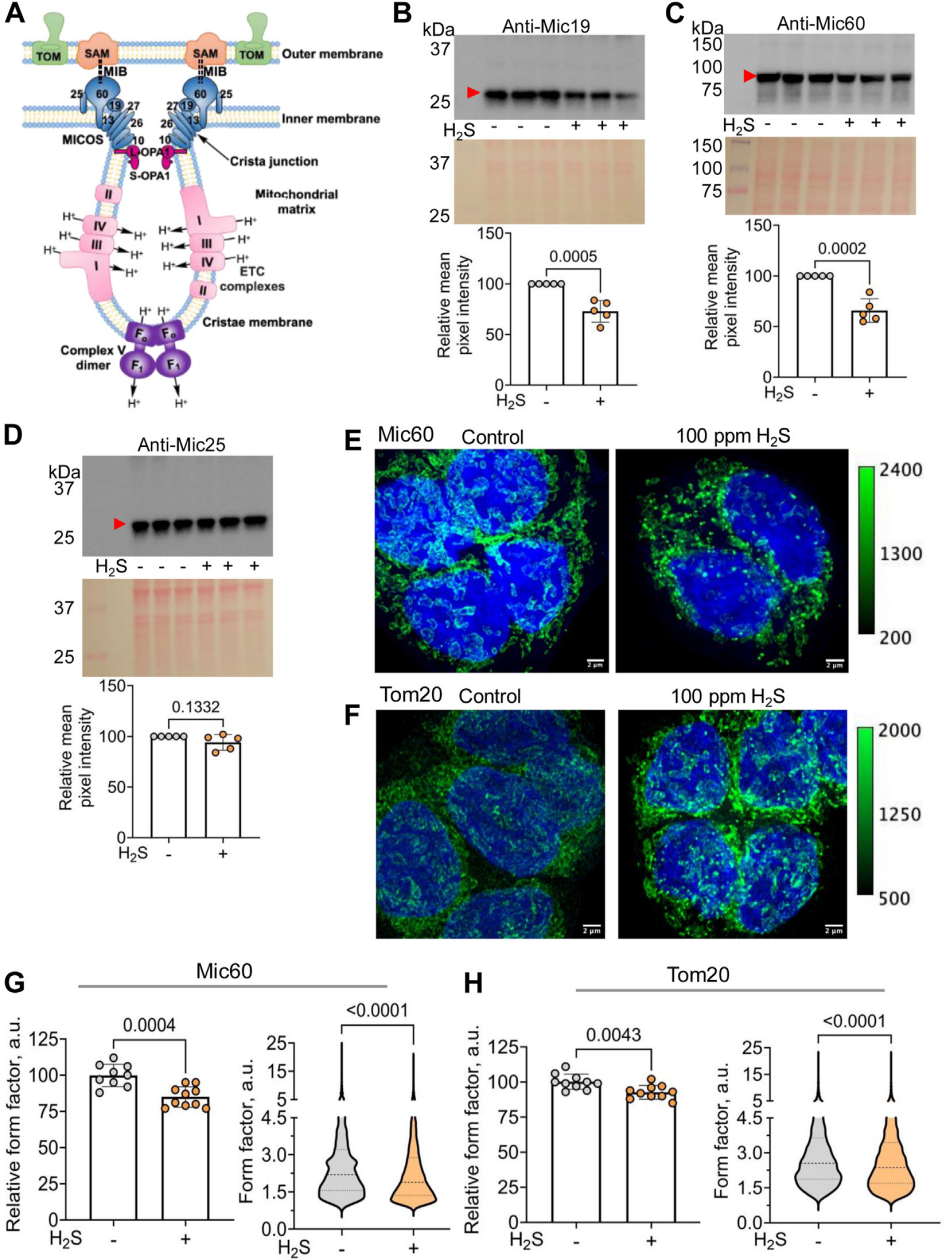
**H_2_S induces cristae loss by disrupting MICOS.***A*, scheme showing the organization of the mitochondrial membranes and cristae which was adapted from Ref (44). The MICOS complex subunits Mic10, Mic13, Mic19, Mic25, Mic26, Mic27, and Mic60 (denoted by numbers only) stabilize the cristae junction. MICOS links the inner and outer mitochondrial membranes through bridging interactions with the sorting and assembly machinery (SAM) complex, known as the mitochondrial intermembrane space bridging complex (MIB, dotted lines). The width of cristae junction is maintained by interactions between the long (L-) membrane bound, and short (S-) soluble isoforms of Opa-1. ETC complexes reside on the flanks and the F_1_F_o_ ATP synthase at the base of cristae, causing curvature. *B*–*D*, Western blot analysis reveals that Mic19 and Mic60, but not Mic25, levels are significantly decreased in HT-29 cells exposed to H_2_S (100 ppm, 24 h). Ponceau *red* staining for equal loading (*middle panels*) and quantitative analysis of the blots (*lower panels*) are shown. Each symbol represents the average of three technical replicates from five independent experiments. *E* and *F*, representative immunofluorescence staining of Mic60 (*E*) and Tom20 (*F*) and DAPI in HT-29 cells cultured ± 100 ppm H_2_S for 24 h (scale bar: 2 μm). The calibration bar shows that the pixel intensity is within the detector’s range, *i.e.*, 0 to 4096. *G* and *H*, Relative form factor per image (*left panels*) for Mic60 (n = 9, control; n = 10, H_2_S) (*G*) and Tom20 (n = 10, ± H_2_S) immunofluorescence staining and the form factor (*right panels*) of each mitochondrion quantified in each image from two independent experiments conducted in duplicate. Two-sample unpaired *t* test was performed for all statistical analyses.

MICOS stabilizes cristae junctions, bridging the inner and the outer membranes *via* interactions with SAM (sorting assembly machinery) in the outer membrane (Fig. 2*A*) (44). To further characterize sulfide-induced changes in cristae size and stability, expression of the MICOS subunits Mic19, Mic25, and Mic60 was assessed. Mic19 and Mic25 are essential for docking Mic60, the core assembly and maintenance component of MICOS, and loss of either protein leads to partial MICOS disassembly and irregular cristae morphology (45, 46). Chronic sulfide exposure decreased Mic19 and Mic60, but not Mic25 expression (Fig. 2, *B*–*D*), which was correlated with an increase in isolated mitochondria as revealed by super-resolution immunofluorescence imaging (Fig. 2, *E* and *F*). Form factor analysis indicated disruption in mitochondrial networks, which was greater with the inner mitochondrial membrane marker, Mic60 (15% lower), than the outer membrane marker, Tom20 (7% lower) (Fig. 2, *G* and *H*, *p* = 0.011). These data suggest some selectivity of sulfide for targeting inner mitochondrial membrane proteins.

### H_2_S influences inner membrane fusion by Oma1-dependent cleavage of Opa1

**Figure 3:**
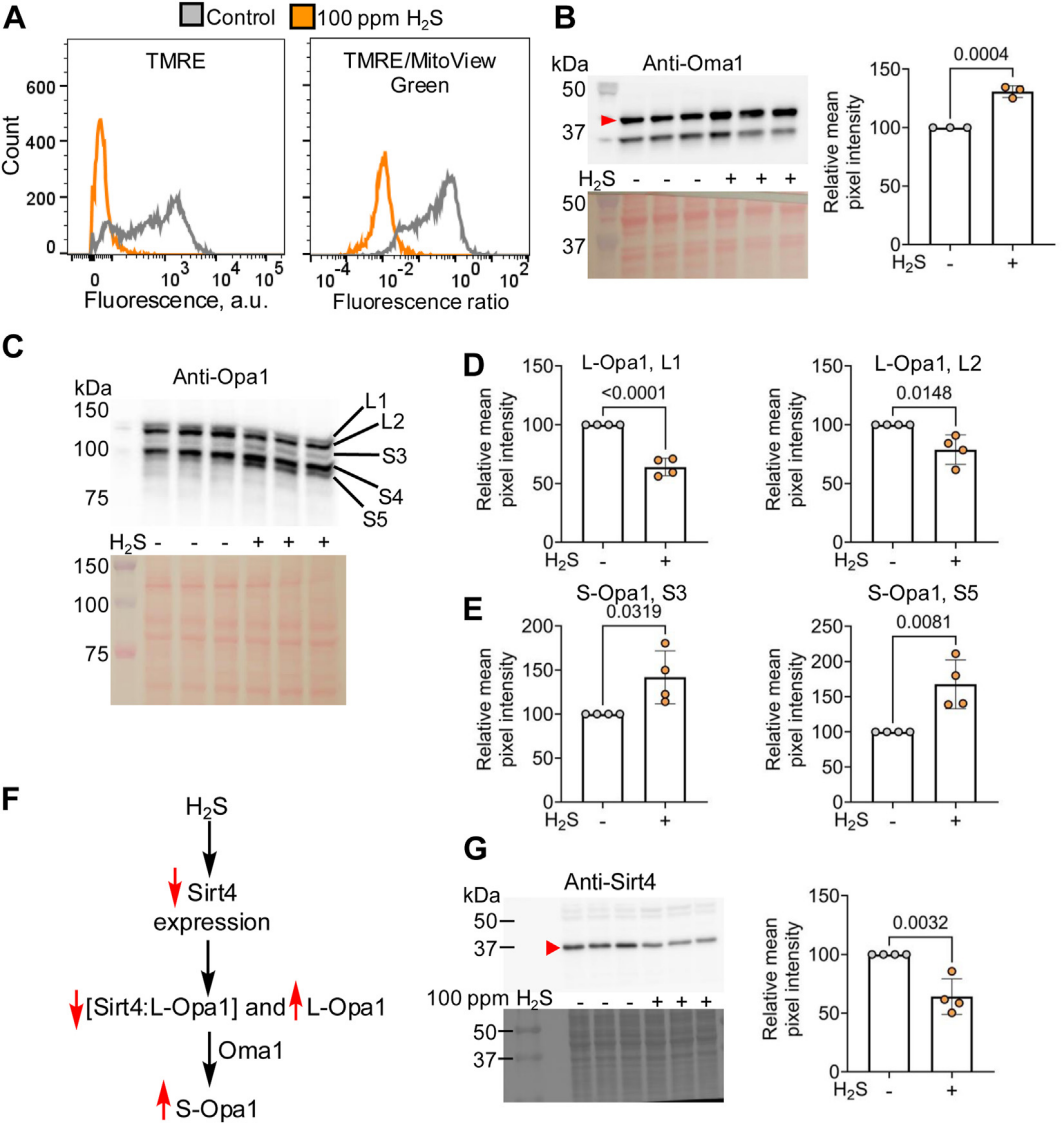
**H_2_S lowers membrane potential and triggers OMA-dependent Opa1 cleavage.***A*, mitochondrial membrane potential in HT-29 cells cultured ±100 ppm H_2_S for 24 h was measured by TMRE staining (*left*) and normalized to mitochondrial mass estimated by MitoView *Green* staining (*right*). *B*, Western blot data (*left*) and quantitative analysis (*right*) shows that H_2_S increases Oma1 expression (n = 4 independent experiments each in triplicate). *C*, H_2_S leads to a redistribution of Opa1 isoforms (L an S denote long and short, respectively). *D* and *E*, quantitation of L1, L2 (*D*), S3, and S5 (*E*) Opa1 isoforms; (n = 4 independent experiments; each data point is the mean of three replicates). *F*, scheme illustrating the possible effect of H_2_S on Sirt4 on L-Opa1 stability. *G*, Western blot analysis of Sirt4 levels (*left*) and quantitation (*right*) in HT-29 cells cultured ± 100 ppm H_2_S. (n = 4 independent experiments conducted in triplicate). A two-sample unpaired *t* test was performed for the statistical analysis and Ponceau staining for equal loading is shown below the respective western blots.

To elucidate the mechanism of sulfide-induced MICOS destabilization, we focused on the Oma1-Opa1 axis. Oma1 stabilizes MICOS (47) while the long and short isoforms of Opa1 regulate cristae junction width and inner membrane fusion (48). We hypothesized that ETC poisoning by H_2_S lowers mitochondrial membrane potential and upregulates and/or activates Oma1, which in turn, enhances the proteolytic conversion of L-to S-Opa1 (9). Sulfide-grown HT-29 cells exhibited a pronounced decrease in mitochondrial membrane potential, which was normalized to mitochondrial mass (Fig. 3*A*), and a small, but significant increase in Oma1 expression (Fig. 3*B*). Furthermore, while the L-Opa1 isoforms decreased, S-Opa1 isoforms increased, indicating enhanced proteolysis (Fig. 3, *C*–*E*). Additionally, expression of Sirt4, the stress-responsive mitochondrial sirtuin that stabilizes L-Opa1 against proteolysis (49), was ∼40% lower in sulfide-grown HT-29 cells (Fig. 3, *F* and *G*). These data are consistent with a model that increased Oma1 and decreased Sirt4 contribute to enhanced L-Opa1 cleavage in sulfide-grown cells. MEF (mouse embryonic fibroblast) cells in which Oma1 was knocked out, exhibited less intense mitochondrial staining with MitoView Green, which as in wild-type cells, increased in response to H_2_S (Fig. S8*A*). Form factor analysis revealed decreased mitochondrial networking in wild-type but not in Oma1-deleted cells in response to H_2_S (Fig. S8*B*).

### H_2_S decreases mitochondrial respiration by disrupting supercomplex stability and assembly

**Figure 4:**
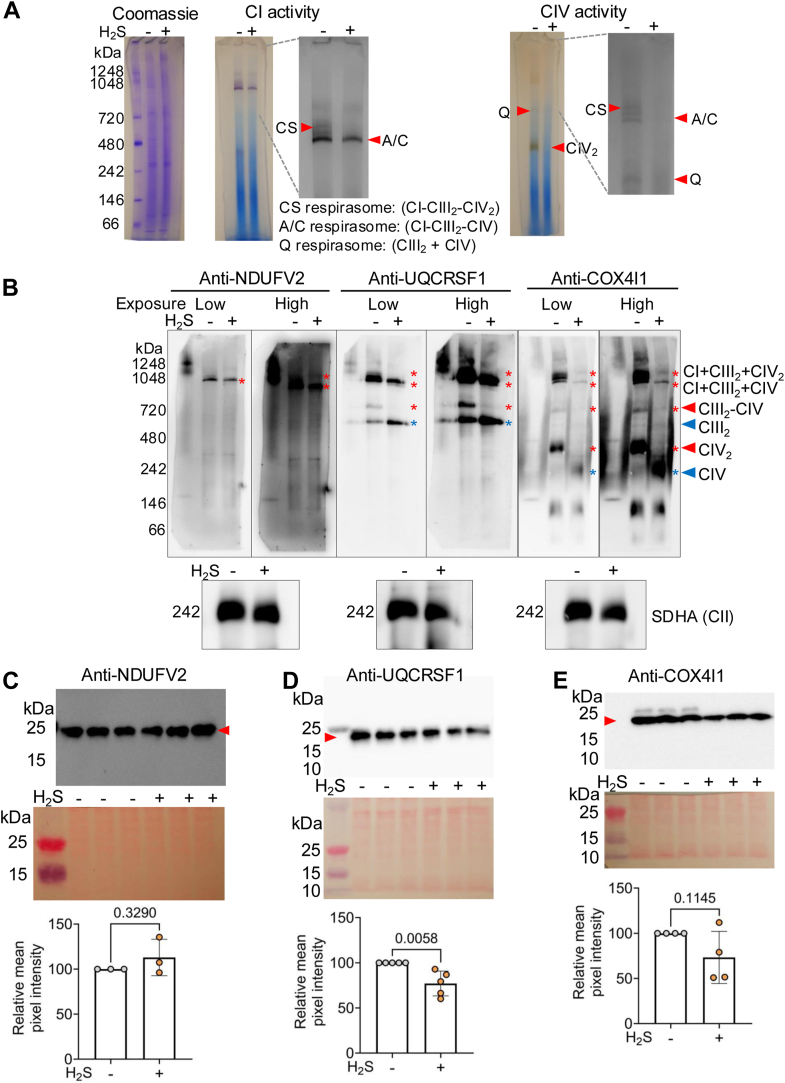
**H_2_S disrupts respirasome and supercomplex stability.***A*, clear native PAGE of mitochondria from HT-29 cells (grown ± 100 ppm H_2_S, 24 h) stained for CI and CIV activity; Coomassie *blue* staining was used to demonstrate equal loading control (*left*). Close-ups of CI and CIV lanes (in *gray* scale) reveal lower activity for CS- and A/C-type respirasomes in H_2_S-treated cells. *B*, whole-cell lysate BN-PAGE and immunoblotting of NDUFV2 (CI), UQCRFS1 (CIII), and COX4I1 (CIV) (displayed at low and high exposure). The *red* and *blue* arrowheads indicate bands that are decreased or increased in abundance in response to H_2_S. Each blot was re-probed with SDHA to show equal loading (*lower*) and is representative of at least three independent experiments. *C*–*E*, SDS-PAGE immunoblots of each protein probed in (*B*) shows that their levels were unchanged in H_2_S-treated cells. Ponceau *red* staining (*middle*) for equal loading, and blot quantitation (*lower*) represent data from 3 to 5 independent experiments each conducted in triplicate. Two-sample unpaired *t* test was used for all statistical analyses.

We examined the effect of sulfide on supercomplexes since cristae morphology affects supercomplex assembly and efficiency (3, 50). Sulfide decreased CI and IV activity associated with high-molecular-weight respirasomes (Fig. 4*A*). A close-up revealed that CI activity was lost from the CS-respirasome (CI-CIII_2_-CIV_2_) but was partially retained in the A/C-respirasomes (CI-CIII_2_-CIV), which cannot be distinguished. A pronounced decrease in CIV activity in the Q-respirasome (CIII_2_-CIV) and the CIV dimer (CIV_2_) was also observed. Blue native PAGE (BN-PAGE) analysis of supercomplex abundance with markers for CI (NDUFV2), CIII (UQCRFS1), and CIV (COX4I1) revealed that CS- and Q-respirasomes and CIV_2_ were severely depleted in H_2_S-grown cells, while the A/C-respirasomes (CI-CIII_2_-CIV) were less impacted (Fig. 4*B*). In contrast, the CIII dimer (CIII_2_) and the CIV monomer (CIV) were markedly increased in intensity in sulfide-grown cells. Each of the marker proteins was separately assessed by SDS-PAGE analysis of whole cell lysates to rule out that differences in their steady-state levels might have contributed to the analysis of supercomplex abundance (Fig. 4, *C*–*E*).

**Figure 5:**
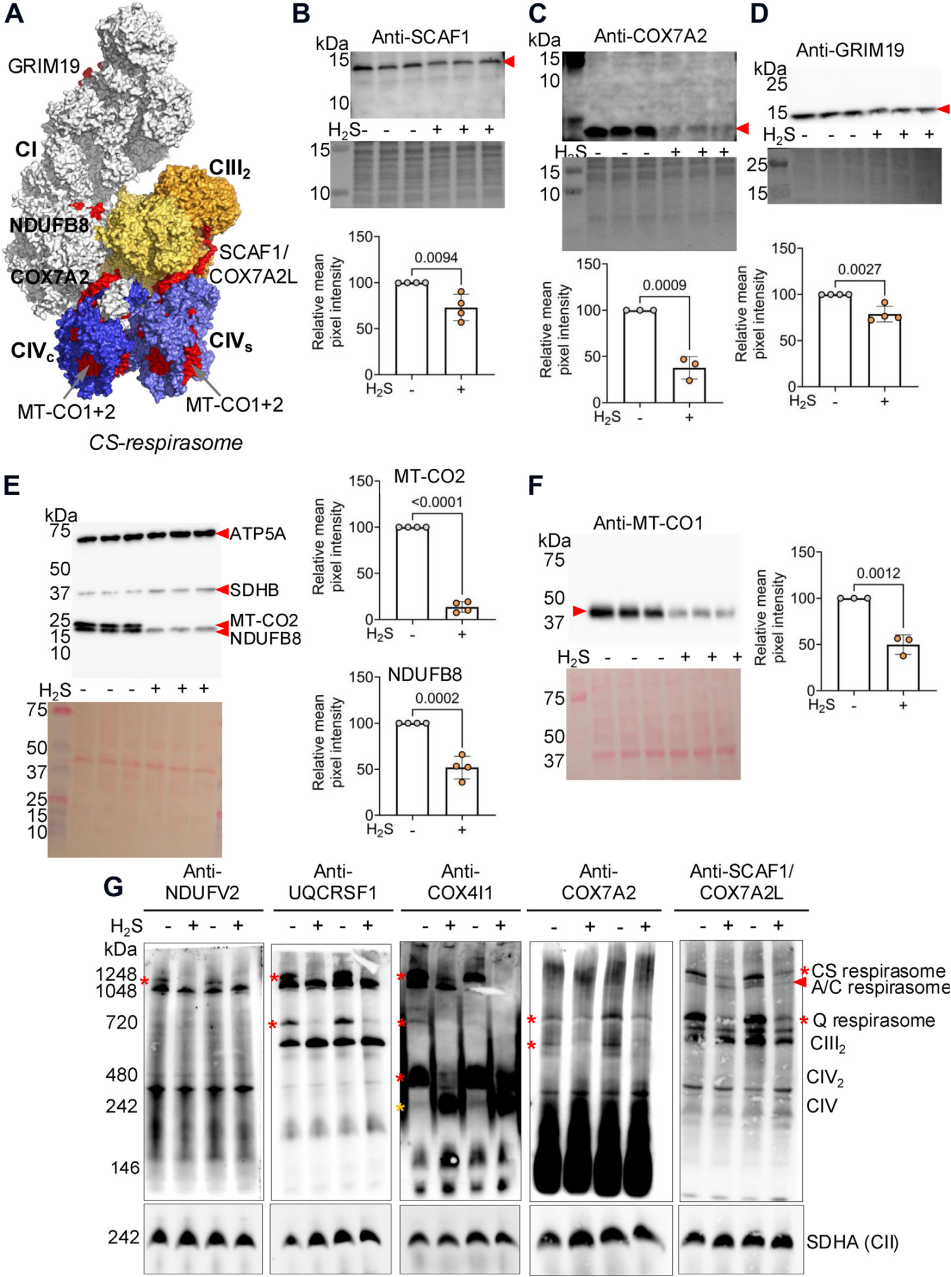
**H_2_S leads to loss of complex I and complex IV scaffold protein from respirasomes.***A*, surface representation of the CS- respirasome (PDB 8PW7) depicting complexes I (*gray*), CIII_2_ (*yellow*) and CIV (*blue*), containing COX7A2 (CIV_c_) or SCAF1/COX7A2L (CIV_s_). The subunits that were probed in *B*–*F* and found to decrease in abundance in response to H_2_S are shown in *red*. *B*–*F*, Western blot analysis of the complex IV scaffold proteins, COX7A2 (*A*) and SCAF1/COX7A2L (*B*), complex I inner membrane anchoring proteins GRIM19 (*C*) and NDUFB8 (*D*), and complex IV core subunits MT-CO2 (*D*) and MT-CO1 (*E*) show significant decreases in H_2_S-grown HT-29 cells (100 ppm, 24 h). Ponceau *red* staining for equal loading quantification are shown for each Western blot. The data are from 3 to 4 independent experiments each conducted in triplicate. A two-sample unpaired *t* test was performed for all statistical analyses. *G*, BN-PAGE immunoblots of HT-29 cell lysates (cultured ± 100 ppm H_2_S, 24 h) shows that SCAF1/COX7A2L (*right panel*) exhibits lower association with the CS-respirasome and with CIII_2_-CIV, while its levels in CIII_2_ is largely unchanged. COX7A2 is less abundant in CIII_2_ and the Q-respirasome. Anti-NDUFV2, UQCRFS1, and are shown side-by-side for verification of protein ID in the SCAF1/COX7A2L immunoblot. *Red* and *yellow* asterisks denote bands that are decreased or increased in response to H_2_S, respectively. The blots were re-probed for SDHA as an equal loading control (*bottom* panels). The data represent mitochondria isolated from two independent experiments.

Scaffold proteins are important for the interactions between CIII and IV in the CS-respirasomes (Fig. 5*A*) (51, 52). Western blot analysis revealed that the CIV scaffold proteins (SCAF1/COX7A2L and COX7A2), CI membrane anchoring proteins (GRIM19 and NDUFB8), as well as the CIV core subunits, MT-CO1 and MT-CO2, were decreased in H_2_S-grown cells (Fig. 5, *B*–*F*). BN-PAGE immunoblot analysis indicated a significant loss of SCAF1/COX7A2L associated with the CS- and Q-respirasomes and loss of COX7A2 associated with the Q-respirasome and CIII_2_ (Fig. 5*G*). The latter assignments were confirmed with antibodies to CI (NDUFV2), CIII (UQCRSF1) and CIV (COX4I1). COX7A2 and NDUFB8, which are significantly lower in H_2_S-grown cells, are associated with the A- and C-respirasomes, while SCAF1/COX7A2L is not involved in their stabilization (Fig. S9).

## Discussion

Mitochondria are hubs for inter-organellar communication *via* physical contact sites with the endoplasmic reticulum, lysosomes, peroxisomes, and lipid droplets (53), and mitochondrial plasticity is a key component of cellular adaptation to stresses and changing metabolic needs (54). The mitochondrion has emerged as a prominent signaling hub for H_2_S, due to its twin potential to promote and inhibit ETC flux. In the gut, where host exposure to microbial-derived H_2_S is generally accepted to be high, the effective exposure of colonocytes, which are sheathed by a mucus layer, as well as the range of diurnal fluctuation in luminal H_2_S is not known. To circumvent the limitations of bolus H_2_S treatment, which leads to its rapid disappearance from the cell culture medium (t^1^/_2_ of 3–4 min at 37 °C) (29), we used a previously described custom-built sulfide growth chamber (40) for chronic delivery of 100 ppm H_2_S gas (corresponding to a dissolved sulfide concentration of 20 μM) over a 24 h period. While insights into metabolic signaling, originating from the interaction of H_2_S with CIV, are beginning to emerge, little is known about how they are linked to mitochondrial ultrastructural changes. In this study, we demonstrate that chronic low-level H_2_S exposure is a significant modifier of mitochondrial ultrastructure and networking and delineates inner membrane protein targets of H_2_S. We posit that the observed effects originate with CIV inhibition by H_2_S, leading to membrane depolarization and a reductive shift in electron carrier pools. The downstream pleiotropic effects are mediated in part by promoting Oma1-dependent cleavage of Opa1, disrupting MICOS stability, and impairing supercomplex assembly. The impacts on mitochondrial networks and respiration are reversible under our experimental conditions, consistent with an adaptive response rather than progression along a cell death pathway.

TEM images revealed that H_2_S induced a significant increase in vesicular ultrastructure, a key indicator of cristae unfolding and mitochondrial swelling (Fig. 1*D*). Greater variability in mitochondrial size was observed in H_2_S-grown *versus* control cells (Figs. 1*D* and S5), which was correlated with a decrease in mitochondrial networking observed by live cell imaging of HEK293, HT-29, HT1080, and SW480 cells (Figs. S2–S4). Levels of Mic60 and Mic19, two of three MICOS components examined in this study, were downregulated by H_2_S (Fig. 2, *B* and *C*). Super-resolution immunofluorescence imaging of Mic60 revealed a substantial decrease in form factor, a measure of mitochondrial networking (41), consistent with decreased inner membrane fusion (Fig. 2, *E* and *G*).

Mic19 is a redox-regulated scaffold protein that associates with Mic60 in its disulfide form (11) and stabilizes the bridging interaction between MICOS in the inner, and Sam50 in the outer mitochondrial membranes (55). Mic19 knockdown increases mitochondrial fragmentation, lowers MT-CO2 expression, and increases vesicular ultrastructure (45), while Mic19 disulfide reduction leads to similar, albeit less pronounced changes (11). ETC inhibition by H_2_S leads to a reductive shift in redox cofactor pools (27, 28) and in cytochrome c (25), which are predicted to disrupt oxidative protein folding in the mitochondrial intermembrane space (Fig. 1*A*), impacting the stability of MICOS and other redox-sensitive proteins. Future experiments that directly evaluate redox changes in target proteins associated with H_2_S-dependent destabilization of MICOS and the respirasome, will provide mechanistic insights into this process.

Mitochondrial flickering, *i.e.* pulsatile inner membrane depolarization, is regulated by the copper transport function of Slc25a3, a mitochondrial copper/phosphate carrier protein (56). Copper-dependent CIV (or cytochrome c oxidase) activity promotes flickering, which is attenuated by the inhibitor, sodium azide. We posit that H_2_S might be an endogenous modulator of mitochondrial flickering *via* CIV inhibition. We demonstrated that H_2_S induces membrane depolarization, which is a known trigger for Oma1 activation and consequent Opa1 proteolysis, a regulator of inner membrane fusion (48, 57) (Fig. 3, *A*–*E*). Opa1 proteolysis could be further augmented by an H_2_S-dependent decrease in Sirt4, which helps stabilize L-Opa1 (49) (Fig. 3, *F* and *G*). In cells lacking Oma1, sulfide failed to elicit a significant effect on mitochondrial networking (Fig. S8).

Structural changes in the mitochondrial inner membrane help orchestrate mitochondrial energy metabolism and fine-tune supercomplex assembly (58). Defects in MICOS and a shift to short Opa1 isoforms have been shown to decrease supercomplex stability and decrease mitochondrial function (1), consistent with our findings that mitochondria in sulfide-grown cells exhibited diminished CI and IV activities associated with the CS- (CI-CIII_2_-CIV_2_) and Q- (CI-CIII2) respirasomes while the A/C-respirasomes were less affected (Fig. 4*A*). The CIV core subunits, MT-CO1 and MT-CO2, which were modestly decreased by acute H_2_S exposure (100 μM, 4 h) (26), were significantly diminished by chronic exposure (∼20 μM, 24 h). Additionally, depletion of CI (GRIM19, NDUFB8), CIII (SCAF1/COX7A2L) and CIV (COX7A2) subunits was observed in H_2_S-grown cells (Fig. 5, *B*–*F*). COX7A2 is a component of CIV_c_, while SCAF1/COX7A2L is a component of CIV_s_ (51), and depletion of either is predicted to destabilize the CS-respirasome (Fig. 5*A*). This prediction was confirmed by the loss of SCAF1/COX7A2L from supercomplexes in which CIV is a component but not from the CIII_2_ dimer (Fig. 5*G*). This conclusion is supported by the depletion of the CS-respirasome and near complete loss of Q-respirasome (Fig. 4*B*), which require SCAF1/COX7A2L for stability (52). Sulfide-induced destabilization of MT-CO1 and MT-CO2 expression is consistent with disrupted mitochondrial bioenergetics and further highlights the link between loss of respiratory supercomplexes and impaired energy metabolism (Fig. S6). We posit that the destabilization of supercomplexes could underlie the variable utilization of oxygen *versus* fumarate as a terminal electron acceptor by tissues (59, 60) and is governed by local differences in H_2_S as well as oxygen concentration.

In summary, our study reveals the potential for sulfide to modulate mitochondrial architecture and function, with possible metabolic implications for other organelles that communicate with this cellular energy hub. The observed changes in mitochondrial ultrastructure and network could be relevant in the pathology of diseases associated with elevated sulfide levels, *e.g.*, inflammatory bowel disease (61, 62), as well as hereditary disorders that impair sulfide oxidation (37, 38). Our data suggest the possibility that at the low concentrations found in most cells, sulfide could be an endogenous regulator of mitochondrial dynamics possibly *via* inhibition of mitochondrial flickering-driven fusion.

## Experimental procedures

### Materials

#### Cell lines

Human colorectal adenocarcinoma HT-29 and human embryonic kidney HEK293 cells were obtained from the American Type Culture Collection (ATCC). *Oma1*^−/−^ MEFs (12) and mito-RFP expressing human colorectal adenocarcinoma HT1080 and SW480 cells were generated as described previously (63).

#### Reagents

Na_2_S, nonahydrate (99.99% purity, (431,648), protease inhibitor cocktail for mammalian tissue extract (P8340), dimethyl sulfoxide (D2650), reduced β-nicotinamide adenine dinucleotide (N8129), and cytochrome c from bovine heart (C2037–500 mg) were from Sigma/Millipore. Dulbecco’s modified Eagle’s medium (DMEM) (with 4.5 g/L glucose, 584 mg/L glutamine, and 110 mg/L sodium pyruvate, (11,995–065)), RPMI 1640 with glutamine (11,875–093), RPMI + HEPES (22,400,105), phenol red-free RPMI (11,835,030), fetal bovine serum (FBS, 10,437–028), penicillin/streptomycin mixture (15,140–122), 0.05% (w/v) trypsin-EDTA (25,300–054), and PBS (10,010–023) were from Gibco. KCl (7447–40–7) was from Acros Organics and Nonidet P40 substitute (74,385) was from Fluka BioChemika. MitoView Green (70,054) was purchased from Biotium. Methanol (A452–4), Tween20 (BP337500), nitro blue tetrazolium chloride (N6495), tetramethylrhodamine ethyl ester perchlorate (TMRE) (T669), and NativePAGE 3 to 12% gels (10-well, BN1001BOX; 15-well, BN1003BOX), and the NativePAGE Sample Prep Kit (BN2008), containing NativePAGE 4x Sample Buffer or pinky lysis buffer (BN2003) and NativePAGE 5% G-250 Sample Additive (BN2004) were purchased from Fisher. EGTA (33,963) was from Cayman Chemical. 10% Pre-cast tris-glycine gels (4,561,033), PVDF membranes (162–0177), thick blot filter paper (1,703,932), Clarity ECL and Clarity Max ECL substrates (1,705,061 and 1,705,062, respectively) were purchased from Bio-Rad. Polystyrene round-bottom tubes (5 ml) with cell-strainer caps used for FACS analysis were from BD Biosciences (720,035). High glass bottom dishes (35 mm, 81,158) for microscopic imaging of live cells were purchased from Ibidi. A propidium iodide flow cytometry kit (ab139418) for DNA staining and FACS analysis was purchased from Abcam. Cylinders with H_2_S in N_2_ (500 L, “A33” 5000 ppm H_2_S with 1% accuracy; #3130) or breathing air containing 5% CO_2_ were from Cryogenic Gases (Detroit, MI, USA).

Reagents for TEM sample preparation were purchased from Electron Microscopy Sciences, including 3% glutaraldehyde and 3% paraformaldehyde in 0.1 M cacodylate buffer (15,950), 0.1 M cacodylate buffer (11,654), 1.5% potassium ferrocyanide in 0.1 M cacodylate buffer (19,150), osmium tetroxide in cacodylate buffer (25,154–10), 0.1 M acetate buffer (11,482–42), 2% uranyl acetate in 0.1 M acetate buffer (22,400–4), 200 proof ethanol (15,055), HPLC grade acetone (022,928.K2), and Spurr’s Resin (14,300).

#### Antibodies

Anti-Grim19 (1:1000; ab110240), anti-MT-CO1 (1:1000; ab14705), anti-MT-CO2 (1:1000; ab110258), anti-NDUFV2 (1:1000; AB183715), anti-OxPhos (1:1000; ab110411), and anti-UQCRFS1 (1:1000; ab14746) were from Abcam. Anti-COX4I1 (1:1000; VPA00544) was purchased from Bio-Rad, and anti-Opa1 (1:1000; 80,471) was purchased from Cell Signaling Technology. Anti-COX7A2 (1:200; 18122-1-AP), anti-COX7A2L/SCAF1 (1:500; 11416-1-AP), anti-Mic19/CHCHD3 (1:5000; 25625-1-AP), anti-Mic25/CHCHD6 (1:2000; 20639-1-AP), anti-Mic60/Mitofilin (1:5000; 10179-1-AP), and anti-Sirt4 (1:1000; 66543-1-IG) were purchased from ProteinTech. Anti-Oma1 (1:1000; SC515788) was purchased from Santa Cruz Biotechnology. Anti-Tom20 (1:1000; MABT160) was purchased from Sigma/Millipore. Anti-rabbit horseradish peroxidase-linked IgG and anti-mouse IgG, horseradish peroxidase-linked antibodies (1:10,000; NA944 V and NA931, respectively) were from GE Healthcare.

### Cell culture

HT-29 cells were maintained in RPMI 1640 medium. HEK293, HT1080, SW480, and MEF cells were maintained in the DMEM medium. Both RPMI and DMEM media were supplemented with 10% FBS along with 100 units/ml penicillin and 100 μg/ml streptomycin. All cells were maintained at 37 °C with ambient O_2_ and 5% CO_2_. During culture in the sulfide growth chamber, cells were maintained in the same culture medium but with twice the volume of medium to prevent acidification as described (40).

### Transmission electron microscopy

Replicate 10 cm plates were seeded with 1 × 10^7^ HT-29 cells and cultured overnight. The following day, fresh 20 ml medium per plate was added, and cells were cultured for 24 h ± 100 ppm H_2_S. Cells were fixed in 3% glutaraldehyde and 3% paraformaldehyde in 0.1 M cacodylate buffer (CB, pH 7.2) overnight at 4 °C. Then, cells were washed 3 × 15 min with CB, and subjected to osmification with 1.5% K_4_Fe(CN)_6_ + 2% OsO_4_ in 0.1 M CB for 1 h at room temperature, followed by 3 × 5 min washes with 0.1 M CB buffer at room temperature. The cells from replicate plates were scraped, combined, and centrifuged at 300 rpm for 10 min. Prewarmed 4% agarose was added to the cell pellets and cooled on ice. Next, the samples were washed 3 × 5 min with 0.1 M sodium acetate buffer (pH 5.2) at room temperature, then stained for 1 h stain with 2% uranyl acetate in 0.1 M sodium acetate buffer (pH 5.2). After 2 × 5 min additional 0.1 M sodium acetate buffer washes and a 1 × 5 min distilled water wash, the samples were dehydrated by serial 15 min washes in ethanol (30, 50, 70, 80, 90, 95, and 100%) before washing in acetone and infiltrating with Spurr’s resin with 1, 2, and 16 h room temperature treatments with 2:1, 1:1 and 1:2 acetone:Spurr’s Resin mixtures, respectively. Next, the samples were placed in 100% Spurr’s resin for 24 h at room temperature before embedding in the resin to polymerize at 70 °C for 24 h. The resulting blocks were then sectioned at 70 nm thickness using a Leica EM UC7 ultramicrotome and imaged at 60 kV with a 0.96 ms exposure using a JEOL 1400+ transmission electron microscope under 2500x magnification with 377 pixels per μm. Mitochondrial ultrastructure classification was done by blinding all images, and then manually annotated with the following classifications as described previously (64): normal, normal-vesicular, vesicular, swollen-vesicular, swollen, and swollen with cristae. Mitochondria (353) were analyzed from 28 control cells, and 427 mitochondria were analyzed from 46 H_2_S cultured cells.

### SDS-PAGE Western blot analysis

#### Sample preparation

Cells were seeded at 2 × 10^6^ cells per 6 cm plate and cultured overnight before replacing with 8 ml fresh cell culture medium and culturing for 24 h ± 100 ppm H_2_S in the sulfide growth chamber as described (40). To harvest, cells were washed once with 5 ml PBS followed by the addition of 0.5 ml 0.05% trypsin for 5 min at 37 °C before collection in 1 ml RPMI medium. Cell suspensions were centrifuged at 1600*g* for 5 min, the pellet was washed with 1 × 1 ml ice-cold PBS, resuspended in 300 μl non-denaturing lysis buffer with protease inhibitor (0.5% (v/v), Nonidet P40 substitute, 25 mM KCl, 20 mM HEPES, pH 7.4), and then frozen until use.

#### Development of SDS-PAGE western blots

Frozen cell pellets were lysed by three freeze-thaw cycles and centrifuged at 13,000*g* for 10 min. Protein content in the supernatant was determined using the Bradford reagent (Bio-Rad). Cell lysates (10–30 μg) were electrophoresed using precast Bio-Rad 10% tris-glycine SDS gels, transferred to PVDF membranes, blocked for 1 h with 5% milk in Tris-buffered saline with 0.3% Tween 20 (TBST). Membranes were soaked overnight in TBST, 5% (w/v) milk containing diluted antibodies, and then quickly washed twice with TBST followed by 4 × 10 min washes with TBST. Membranes were exposed for 90 min to the secondary antibody (horseradish peroxidase-linked anti-rabbit or anti-mouse IgG used at a 1:10,000 dilution in TBST, 5% milk). The membranes were quickly washed twice with TBST followed by 5 × 10 min TBST washes and two rinses with TBS before treating with clarity ECL substrate (Bio-Rad). Signals were detected using a Bio-Rad ChemiDoc Imaging System, capturing 15.5 × 12.4 cm images. All images were exported as 16-bit TIF files and imported into Fiji (65) for semi-quantitative analysis, which was performed by drawing equal-sized rectangles over each band to estimate its mean pixel intensity. The background was subtracted from each band.

### Native gel electrophoresis of mitochondrial proteins

#### Crude mitochondrial isolations

P2 crude mitochondrial fractions were isolated as previously described with modifications (66). Cells were seeded at 5 to 6 × 10^6^ cells per 10 cm plate in triplicate and cultured overnight before replacing with fresh 20 ml medium and culturing for 24 h ± 100 ppm H_2_S. Cells were harvested by washing each plate once with 8 ml PBS, treating with 1 ml 0.05% trypsin for 5 min at 37 °C before harvesting in 6 ml RPMI medium per plate. Cells were pooled in 50 ml conical tubes, pelleted for 5 min at 1600*g* at 4 °C, washed once with 25 ml ice-cold PBS, and pelleted again for 5 min at 1600*g* at 4 °C. The cell pellet was frozen for later use or resuspended in 3 ml ice-cold CP-1 buffer (100 mM KCl, 50 mM Tris, 2 mM EGTA, pH 7.4 + protease inhibitor) for homogenization on ice by 20 passes with a glass homogenizer with a Teflon tip, followed by 5 passes through a 27-gauge needle using a 5 ml syringe. The homogenate was pelleted at 700*g* for 10 min at 4 °C. The top 90% of the mitochondria-containing supernatant was collected while the pellet was suspended in an additional 3 ml of CP-1 buffer and re-homogenized by 20 additional passes with the glass homogenizer and five additional passes through the 27-gauge needle. The resulting homogenate was pelleted at 700*g* for 10 min at 4 °C, and the top 90% of the second supernatant batch was pooled with the first and aliquoted in 6 × 1.7 ml conical tubes and pelleted at 10,400*g* for 10 min at 4 °C. The resulting mitochondria-enriched P1 pellets were consolidated in 3 × 1.7 ml conical tubes using 3 ml of CP-1 buffer and centrifuged again at 10,400*g* for 10 min at 4 °C to obtain crude P2 pellets. The latter were combined into one slurry using 450 μl CP-1 buffer, which was pipetted up and down with a 200 μl pipet before using 5 μl of the mitochondrial slurry to determine protein content, using the Bradford reagent (Bio-Rad) and a calibration curve generated with 0.5 to 4 mg/ml BSA standards in CP-1 buffer. The mitochondria were then aliquoted in 100 μg fractions for later use and stored at −80 °C.

Before use, 100 μg aliquots of P2 mitochondria were reisolated by thawing on ice and then centrifuged at 12,600*g* for 5 min. The supernatant was removed and the pellet was resuspended in 54 μl 1x pink lysis buffer (Fisher) + Sigma protease inhibitor cocktail. Next, 12 μl of 5% digitonin (Sigma) was added to achieve a ratio of protein/detergent of 1:6, the samples were mixed by pipetting, and incubated on ice for 15 min before centrifugation for 20 min at 20,000*g*. The top 60 μl of supernatant, which contained solubilized mitochondrial proteins, was transferred into a pre-labeled tube. Next, 4 μl of blue native loading buffer (Invitrogen, BN20041) was added, yielding a final concentration of 1.4 μg/μl in each sample for loading in precast Bis-Tris 3 to 12% gradient gels (Invitrogen, BN2011 B × 10) for either clear native (CN) or blue native (BN) PAGE.

#### Preparation of whole cell lysates

Digitonin permeabilized cell lysates for BN-PAGE analysis were prepared as previously reported with slight modifications (67). Cells were seeded at 5 to 6 × 10^6^ cells per 10 cm plate in quadruplicate and cultured overnight before replacing with 20 ml fresh culture medium and placing them in a growth chamber for 24 h ± 100 ppm H_2_S. Cells were harvested by washing each plate with 8 ml PBS before treating with 1 ml 0.05% trypsin for 5 min at 37 °C. The trypsinized cells were collected in 7 ml RPMI medium/plate and centrifuged at 1600*g* for 5 min. The cell pellet was suspended in 8 ml ice-cold PBS and a 100 μl aliquot was diluted 1:1 (v/v) with trypan blue for cell counting. An aliquot (20 μl) was used for counting in a Cellometer (Nexelcom). The remaining cell suspension was centrifuged at 1600*g* for 5 min in several 1.7 ml conical tubes to obtain 2.5 × 10^6^ cell aliquots; each was washed once with 1 ml ice-cold PBS, resuspended in 200 μl ice-cold PBS, and then spiked with 7.5 μl of 5% digitonin to yield a final concentration of 1.8 mg/ml digitonin. Cells were mixed by pipetting and incubated on ice for 10 min before adding 1 ml ice-cold PBS and spinning for 5 min at 20,000*g*. The supernatant was aspirated, and cells were washed with 1 ml ice-cold PBS before centrifuging for 5 min at 20,000*g*. The resulting cell pellets, which are permeabilized cells, were frozen at −80 °C until later use.

Before use, three aliquots of isolated, permeabilized whole cell lysates were thawed on ice for 30 min, consolidated in 310 μl of pink lysis buffer + protease inhibitor. The protein content of the samples was measured by the Bradford assay, and ∼310 μg protein was spiked with 25 μl of 5% digitonin (w/v) to a final protein/detergent ratio of 1:4. The samples were mixed by pipetting and then incubated on ice for 10 min before centrifuging for 30 min at 20,000*g*. The top 270 μl of the supernatant was transferred to pre-labeled tubes containing 20 μl of NativePAGE 5% G-250 Sample Additive (Fisher, BN2004). The final concentration of solubilized protein for BN-PAGE was ∼0.9 μg/μl.

#### Development of BN-PAGE Western blots

Solubilized mitochondrial proteins were separated by loading 10 μl P2 crude mitochondrial sample (14 μg per lane) or 40 μl of whole cell lysate sample (36 μg per lane) onto precast Bis-Tris 3 to 12% gradient gels (Invitrogen, BN2011 B × 10) in the NativePAGENovex Bis-Tris Gel System according to manufacturer’s recommendations for organelle protocols and following key considerations as described previously (68). Proteins were separated with NativePAGE anode buffer (Invitrogen, BN2001) and dark blue cathode buffer (Invitrogen, BN2002) for one hat 150 V at 4 °C before switching to light cathode buffer (Invitrogen, BN2002) to run overnight at 20 to 30 V at 4 °C. Proteins from the resulting gels were transferred to PVDF membranes (Bio-Rad) using the wet transfer technique with the XCell II Blot Module overnight at 4 °C. After transfer, the membranes were fixed for 5 min in 8% acetic acid, washed twice with water for 5 min, and then clipped to air dry. Next, each blot was rinsed with methanol (3 × 5 min) and water (3 × 5 min), and blocked in 5% milk in TBST for 30 min before blotting with the indicated primary antibodies according to the manufacturers’ recommendations. Secondary anti-mouse HRP antibodies were the same as listed for the SDS-PAGE analysis, but BioRad Clarity Max (1,705,062) was used as the ECL substrate to visualize the native PAGE immunoblotted bands.

### CN-PAGE in-gel activity assays

The in-gel activity assays were performed as previously described (68), by loading 20 μg (1.4 μg/μl) of solubilized P2 crude mitochondria in 10-well, 3 to 12% Bis-Tris gradient gels in the NativePAGE Novex Gel system described above, but by first separating with NativePAGE anode buffer and light blue cathode buffer for 30 min at 150 V before switching to clear cathode buffer to avoid excessive blue color of the Coomassie dye on the gel that would interfere with the color of the activity. The gel was run for an additional 2.5 to 3 h at 250 V for better separation of the SC bands (68). Complex I activity was stained with 0.1 mg/ml NADH (2 mg) and 2.5 mg/ml nitrotetrazolium blue chloride (50 mg) in 20 ml of 2 mM Tris-HCl, pH 7.4 for 20 to 30 min before stopping the reaction with 10% acetic acid, rinsing with water, and then imaging (68). Complex IV activity was stained with 2 mg/ml diaminobenzidine, 4 mg/ml bovine cytochrome c in 45 mM phosphate buffer, pH 7.4 for an hour before stopping the reaction with 10% acetic acid, rinsing the gel with water, and imaging (68).

### Mitochondrial networking analysis

#### MitoView green-stained cells

HT29, HEK293, MEF, and MitoTag-RRP expressing HT1080 and SW480 cells were plated at 50,000 to 100,000 cells per 35 mm high glass bottom dishes (Ibidi, 81,158) and cultured overnight before replacing the medium and placed in the growth chamber for 24 h ± 100 ppm H_2_S. To stain HT29, HEK293, and MEF cells, the culture medium was replaced with serum-free RPMI containing 50 nM MitoView green added from a 200 μM stock in DMSO. The cells were stained in the growth chamber for 30 min before washing the cells twice with serum-free RPMI and replacing the medium with phenol red-free RPMI + FBS with penstrep and cultured for an additional 60 to 90 min before imaging. The MitoTag-RRP expressing cells had been previously validated to have mitochondrial localization of RFP and were not stained (63). Cells were imaged on a Nikon X1 Yokogawa Spinning Disk Confocal microscope with CO_2_ and temperature regulation, 63x oil objective with a 1.49 numerical aperture, equipped with an Andor DU-888 monochrome camera for widefield imaging and a 488 nm laser for exciting the MitoView Green dye or a 561 nm laser for exciting mitoRFP. The pixel sizes per experiment ranged from 110 to 217 nm with resolution between 208 to 217 nm, depending on the experiment.

#### Image analysis and form factor calculations

The form factor (a function of both mitochondrial length and branching, equal to perimeter2/4π × area) was assessed to measure mitochondrial networking using FIJI as described previously (69). For this, maximal intensity projections of the fluorescence intensity images were made, and equal intensity thresholding was used for all analyzed images. The threshold images were used to identify mitochondria, whose area and perimeter were exported into an excel file to calculate the form factor for each mitochondrion. The mitochondrial data were exported in bulk to quantify the form factor of every identified mitochondrion, or each image’s data was averaged per image to quantify the average form factor per image. The data were used to generate violin plots in Prism.

### Immunofluorescence microscopy

Cells (25,000–50,000 cells/well in a 24-well plate) were seeded in pre-sterilized (190 proof ethanol, 10 min), poly-d-lysine precoated (1 h, 37 °C) coverslips and cultured for 24 h. The next day, the medium was changed, and cells were cultured for 24 h ± 100 ppm H_2_S before fixation with 4% paraformaldehyde for 15 min. Cells were washed with PBS, permeabilized with 0.25% Triton X-100 in PBS for 5 min, blocked in CST blocking buffer (5% Donkey/Goat serum/BSA, 0.3% Triton X-100 in PBS) for 30 min, and then incubated for 1 h with primary antibodies against Mic60 or Tom20 diluted 1:200 in buffer (1% BSA, 0.3% Triton X-100 in PBS). The samples were washed 3 × 5 min with 1% BSA, 0.3% Triton X-100 in PBS before treating with secondary 488 Donkey/Goat-anti-rabbit antibody (Invitrogen) diluted to 1:500 in buffer (1% BSA, 0.3% Triton X-100 in PBS) for an hour at room temperature in a humidified chamber. The samples were washed 3 × 5 min with 1% BSA, 0.3% Triton X-100 in PBS before letting the coverslips dry before mounting them on glass slides using ProLong Gold + DAPI (Fisher) and imaged using a Zeiss LSM 980 Airyscan two microscope and detector equipped with a 63x oil objective with a 1.4 numerical aperture, a pixel size of 65.7 nm, and an anticipated resolution of 107 nm using the Airyscan SR mode. Post-processing was done with Zen 3.4 (Blue edition), and maximum intensity projections and segmenting of mitochondria for form factor analysis were done in FIJI.

### Flow cytometry analysis

FACS analysis was conducted using the Bio-Rad Ze5 multi-laser, high-speed cell analyzer operated with the Everest software package at the University of Michigan Flow Cytometry Core Facility. All data were analyzed using FlowJO (v10.8.1).

#### TMRE membrane potential measurements

HT-29 cells were seeded at 500,000 cells per well in 6-well plates and grown overnight before replacing the medium and placing cells in the growth chamber ± 100 ppm H_2_S for 24 h. The medium was replaced with serum-free RPMI containing 50 nM TMRE and 50 nM MitoView Green (in DMSO) and staining was continued for 30 min ± 100 ppm H_2_S culture, before washing the cells twice with room temperature RPMI and replacing the medium with phenol red-free RPMI + FBS with penstrep. The cells were cultured in this medium for an additional hour before harvesting to minimize nonspecific binding of the dyes. Then, the medium was aspirated, and cells were collected by scraping in 500 μl ice-cold PBS, suspended by pipetting, and filtered through 5-mL BD round bottom falcon tubes with cell-strainer caps, for FACS analysis. FlowJO (v10.8.1) was used to conduct gating and ratio TMRE:MitoView Green fluorescence for each analyzed cell and to plot histograms of the raw and ratioed TMRE fluorescence.

#### Cell cycle analysis

To evaluate the reversibility of H_2_S-induced changes in the cell cycle, 2 × 10^6^ HT-29 cells per 6 cm plate (in duplicate) were cultured overnight. The next day, fresh 8 ml medium was added to each plate before placing them in the growth chamber ± 100 ppm H_2_S for 24 h. Then, cells were either harvested or allowed to recover in the absence of H_2_S for 24 to 48 h with fresh medium changes every 24 h. The conditioned medium was collected in 15 ml conical tubes and cells were washed with PBS, which was collected in the same tube. Next, the cells were treated with 0.5 ml 0.05% trypsin for 5 min at 37 °C and harvested with 1 ml RPMI medium, pooled with the conditioned medium and PBS wash, and centrifuged at 500*g* for 5 min at 4 °C. Cells were washed once with 1 ml ice-cold PBS by gentle suspension and subsequent cell pelleting at 500*g* for 5 min at 4 °C. Cells were then fixed in 66% ethanol on ice by suspending the cell pellet in 400 μl of ice-cold PBS and slowly adding 800 μl of ice-cold absolute ethanol, which was mixed by pipetting. The fixed cells were stored at 4 °C until all time points were harvested before proceeding with DNA staining and FACS analysis with the propidium iodide flow cytometry kit for cell cycle analysis (ab139418 from Abcam), following the manufacturer’s protocols.

### Oxygen consumption rate (OCR) measurements

All OCR measurements were performed on the Oroboros Instruments Corp respirometer at 37 °C with a stirring rate of 750 rpm. Cells were harvested from 10 cm plates, and washed with 1 × 8 ml PBS prior to digestion with 1 ml trypsin (0.05%) at 37 °C for 5 min. Cells were collected in 7 ml of the cell culture medium and centrifuged at 1600*g* for 5 min, and pellets were resuspended in 1 ml modified PBS (MPBS) or DPBS + 5 mM glucose + 20 mM HEPES, pH 7.4. Cell suspensions were transferred to pre-weighed tubes, centrifuged at 1600*g* for 3 min, and the supernatant was carefully aspirated with a 2 μl tip fixed to a vacuum line. The wet weight of the pellet was determined to prepare 5% (w/v) cell suspensions in MPBS, which were kept on ice and used for dilution to 1% (w/v) suspensions in MPBS for OCR experiments.

Analysis of OCR traces was performed using DatLab v6 (Oroboros Instruments, Austria) and replotted in Origin 7.0. Recovery time following Na_2_S injection is defined as the time taken by cells to return to a new stationary basal OCR.

### Statistical analysis

Statistical analysis for pairwise comparisons was performed using the two-sample unpaired *t* test.

## Data availability

All data are contained within the manuscript.

## Supporting information

This article contains supporting information.

## Conflict of interest

The authors declare that they have no conflicts of interest with the contents of this article.

## References

[1] Cogliati S., Enriquez J.A., Scorrano L. (2016). Mitochondrial cristae: where beauty meets functionality. Trends Biochem. Sci..

[2] Vercellino I., Sazanov L.A. (2022). The assembly, regulation and function of the mitochondrial respiratory chain. Nat. Rev. Mol. Cell Biol..

[3] Cogliati S., Frezza C., Soriano M.E., Varanita T., Quintana-Cabrera R., Corrado M. (2013). Mitochondrial cristae shape determines respiratory chain supercomplexes assembly and respiratory efficiency. Cell.

[4] Lopez-Fabuel I., Le Douce J., Logan A., James A.M., Bonvento G., Murphy M.P. (2016). Complex I assembly into supercomplexes determines differential mitochondrial ROS production in neurons and astrocytes. Proc. Natl. Acad. Sci. U. S. A..

[5] Lapuente-Brun E., Moreno-Loshuertos R., Acin-Perez R., Latorre-Pellicer A., Colas C., Balsa E. (2013). Supercomplex assembly determines electron flux in the mitochondrial electron transport chain. Science.

[6] Moe A., Di Trani J., Rubinstein J.L., Brzezinski P. (2021). Cryo-EM structure and kinetics reveal electron transfer by 2D diffusion of cytochrome c in the yeast III-IV respiratory supercomplex. Proc. Natl. Acad. Sci. U. S. A..

[7] Vercellino I., Sazanov L.A. (2021). Structure and assembly of the mammalian mitochondrial supercomplex CIII(2)CIV. Nature.

[8] Mishra P., Carelli V., Manfredi G., Chan D.C. (2014). Proteolytic cleavage of Opa1 stimulates mitochondrial inner membrane fusion and couples fusion to oxidative phosphorylation. Cell Metab..

[9] Gilkerson R., De La Torre P., St Vallier S. (2021). Mitochondrial OMA1 and OPA1 as gatekeepers of organellar structure/function and cellular stress response. Front. Cell Dev. Biol..

[10] Glancy B., Kim Y., Katti P., Willingham T.B. (2020). The functional impact of mitochondrial structure across subcellular scales. Front. Physiol..

[11] Sakowska P., Jans D.C., Mohanraj K., Riedel D., Jakobs S., Chacinska A. (2015). The oxidation status of Mic19 regulates MICOS assembly. Mol. Cell Biol..

[12] Bohovych I., Dietz J.V., Swenson S., Zahayko N., Khalimonchuk O. (2019). Redox regulation of the mitochondrial quality control protease Oma1. Antioxid. Redox Signal..

[13] Hanna D., Kumar R., Banerjee R. (2023). A metabolic paradigm for hydrogen sulfide signaling via electron transport chain plasticity. Antioxid. Redox Signal..

[14] Kumar R., Banerjee R. (2021). Regulation of the redox metabolome and thiol proteome by hydrogen sulfide. Crit. Rev. Biochem. Mol. Biol..

[15] Filipovic M.R., Zivanovic J., Alvarez B., Banerjee R. (2018). Chemical biology of H_2_S signaling through persulfidation. Chem. Rev..

[16] Mustafa A.K., Gadalla M.M., Sen N., Kim S., Mu W., Gazi S.K. (2009). H_2_S signals through protein S-sulfhydration. Sci. Signal..

[17] Singh S., Banerjee R. (2011). PLP-dependent H_2_S biogenesis. Biochim. Biophys. Acta.

[18] Landry A.P., Roman J., Banerjee R. (2021). Structural perspectives on H_2_S homeostasis. Curr. Opin. Struct. Biol..

[19] Cuevasanta E., Denicola A., Alvarez B., Moller M.N. (2012). Solubility and permeation of hydrogen sulfide in lipid membranes. PLoS One.

[20] Dickhout J.G., Carlisle R.E., Jerome D.E., Mohammed-Ali Z., Jiang H., Yang G. (2012). Integrated stress response modulates cellular redox state via induction of cystathionine gamma-lyase: cross-talk between integrated stress response and thiol metabolism. J. Biol. Chem..

[21] Longchamp A., Mirabella T., Arduini A., MacArthur M.R., Das A., Trevino-Villarreal J.H. (2018). Amino acid restriction triggers angiogenesis via GCN2/ATF4 regulation of VEGF and H_2_S production. Cell.

[22] Kumar R., Vitvitsky V., Sethaudom A., Singhal R., Solanki S., Alibeckoff S. (2024). Sulfide oxidation promotes hypoxic angiogenesis and neovascularization. Nat. Chem. Biol..

[23] Kabil O., Yadav V., Banerjee R. (2016). Heme-dependent metabolite switching regulates H_2_S synthesis in response to ER stress. J. Biol. Chem..

[24] Landry A.P., Ballou D.P., Banerjee R. (2017). H_2_S oxidation by nanodisc-embedded human sulfide quinone oxidoreductase. J. Biol. Chem..

[25] Vitvitsky V., Miljkovic J.L., Bostelaar T., Adhikari B., Yadav P.K., Steiger A.K. (2018). Cytochrome c reduction by H_2_S potentiates sulfide signaling. ACS Chem. Biol..

[26] Hanna D.A., Diessl J., Guha A., Kumar R., Andren A., Lyssiotis C. (2024). H(2)S preconditioning induces long-lived perturbations in O(2) metabolism. Proc. Natl. Acad. Sci. U. S. A..

[27] Libiad M., Vitvitsky V., Bostelaar T., Bak D.W., Lee H.J., Sakamoto N. (2019). Hydrogen sulfide perturbs mitochondrial bioenergetics and triggers metabolic reprogramming in colon cells. J. Biol. Chem..

[28] Vitvitsky V., Kumar R., Diessl J., Hanna D.A., Banerjee R. (2024). Rapid HPLC method reveals dynamic shifts in coenzyme Q redox state. J. Biol. Chem..

[29] Vitvitsky V., Kumar R., Libiad M., Maebius A., Landry A., Banerjee R. (2021). The mitochondrial NADH pool is involved in hydrogen sulfide signaling and stimulation of aerobic glycolysis. J. Biol. Chem..

[30] Carballal S., Vitvitsky V., Kumar R., Hanna D.A., Libiad M., Gupta A. (2021). Hydrogen sulfide stimulates lipid biogenesis from glutamine that is dependent on the mitochondrial NAD(P)H pool. J. Biol. Chem..

[31] Chatzi A., Manganas P., Tokatlidis K. (2016). Oxidative folding in the mitochondrial intermembrane space: a regulated process important for cell physiology and disease. Biochim. Biophys. Acta.

[32] Mishanina T.V., Libiad M., Banerjee R. (2015). Biogenesis of reactive sulfur species for signaling by hydrogen sulfide oxidation pathways. Nat. Chem. Biol..

[33] Vitvitsky V., Kabil O., Banerjee R. (2012). High turnover rates for hydrogen sulfide allow for rapid regulation of its tissue concentrations. Antioxid. Red Signal..

[34] Furne J., Saeed A., Levitt M.D. (2008). Whole tissue hydrogen sulfide concentrations are orders of magnitude lower than presently accepted values. Am. J. Physiol. Regul. Integr. Comp. Physiol..

[35] Macfarlane G.T., Gibson G.R., Cummings J.H. (1992). Comparison of fermentation reactions in different regions of the human colon. J. Appl. Bacteriol..

[36] Deplancke B., Finster K., Graham W.V., Collier C.T., Thurmond J.E., Gaskins H.R. (2003). Gastrointestinal and microbial responses to sulfate-supplemented drinking water in mice. Exp. Biol. Med. (Maywood).

[37] Tiranti V., Viscomi C., Hildebrandt T., Di Meo I., Mineri R., Tiveron C. (2009). Loss of ETHE1, a mitochondrial dioxygenase, causes fatal sulfide toxicity in ethylmalonic encephalopathy. Nat. Med..

[38] Friederich M.W., Elias A.F., Kuster A., Laugwitz L., Larson A.A., Landry A.P. (2020). Pathogenic variants in SQOR encoding sulfide:quinone oxidoreductase are a potentially treatable cause of Leigh disease. J. Inherit. Metab. Dis..

[39] Rumbeiha W.K., Kim D.S., Min A., Nair M., Giulivi C. (2023). Disrupted brain mitochondrial morphology after in vivo hydrogen sulfide exposure. Sci. Rep..

[40] Hanna D.A., Vitvitsky V., Banerjee R. (2023). A growth chamber for chronic exposure of mammalian cells to H(2)S. Anal. Biochem..

[41] Koopman W.J., Visch H.J., Verkaart S., van den Heuvel L.W., Smeitink J.A., Willems P.H. (2005). Mitochondrial network complexity and pathological decrease in complex I activity are tightly correlated in isolated human complex I deficiency. Am. J. Physiol. Cell Physiol..

[42] Picard M., Shirihai O.S., Gentil B.J., Burelle Y. (2013). Mitochondrial morphology transitions and functions: implications for retrograde signaling?. Am. J. Physiol. Regul. Integr. Comp. Physiol..

[43] Mitra K., Wunder C., Roysam B., Lin G., Lippincott-Schwartz J. (2009). A hyperfused mitochondrial state achieved at G1-S regulates cyclin E buildup and entry into S phase. Proc. Natl. Acad. Sci. U. S. A..

[44] Anand R., Reichert A.S., Kondadi A.K. (2021). Emerging roles of the MICOS complex in cristae dynamics and biogenesis. Biology (Basel).

[45] Darshi M., Mendiola V.L., Mackey M.R., Murphy A.N., Koller A., Perkins G.A. (2011). ChChd3, an inner mitochondrial membrane protein, is essential for maintaining crista integrity and mitochondrial function. J. Biol. Chem..

[46] An J., Shi J., He Q., Lui K., Liu Y., Huang Y. (2012). CHCM1/CHCHD6, novel mitochondrial protein linked to regulation of mitofilin and mitochondrial cristae morphology. J. Biol. Chem..

[47] Viana M.P., Levytskyy R.M., Anand R., Reichert A.S., Khalimonchuk O. (2021). Protease OMA1 modulates mitochondrial bioenergetics and ultrastructure through dynamic association with MICOS complex. iScience.

[48] von der Malsburg A., Sapp G.M., Zuccaro K.E., von Appen A., Moss F.R., Kalia R. (2023). Structural mechanism of mitochondrial membrane remodelling by human OPA1. Nature.

[49] Lang A., Anand R., Altinoluk-Hambuchen S., Ezzahoini H., Stefanski A., Iram A. (2017). SIRT4 interacts with OPA1 and regulates mitochondrial quality control and mitophagy. Aging (Albany NY).

[50] Kuhlbrandt W. (2015). Structure and function of mitochondrial membrane protein complexes. BMC Biol..

[51] Vercellino I., Sazanov L.A. (2024). SCAF1 drives the compositional diversity of mammalian respirasomes. Nat. Struct. Mol. Biol..

[52] Calvo E., Cogliati S., Hernansanz-Agustin P., Loureiro-Lopez M., Guaras A., Casuso R.A. (2020). Functional role of respiratory supercomplexes in mice: SCAF1 relevance and segmentation of the Q(pool). Sci. Adv..

[53] Gordaliza-Alaguero I., Canto C., Zorzano A. (2019). Metabolic implications of organelle-mitochondria communication. EMBO Rep..

[54] Chen W., Zhao H., Li Y. (2023). Mitochondrial dynamics in health and disease: mechanisms and potential targets. Signal. Transduct Target Ther..

[55] Ott C., Ross K., Straub S., Thiede B., Gotz M., Goosmann C. (2012). Sam50 functions in mitochondrial intermembrane space bridging and biogenesis of respiratory complexes. Mol. Cell Biol..

[56] Murata D., Roy S., Lutsenko S., Iijima M., Sesaki H. (2024). Slc25a3-dependent copper transport controls flickering-induced Opa1 processing for mitochondrial safeguard. Dev. Cell.

[57] Ge Y., Shi X., Boopathy S., McDonald J., Smith A.W., Chao L.H. (2020). Two forms of Opa1 cooperate to complete fusion of the mitochondrial inner-membrane. Elife.

[58] Bohovych I., Fernandez M.R., Rahn J.J., Stackley K.D., Bestman J.E., Anandhan A. (2015). Metalloprotease OMA1 fine-tunes mitochondrial bioenergetic function and respiratory supercomplex stability. Sci. Rep..

[59] Spinelli J.B., Rosen P.C., Sprenger H.G., Puszynska A.M., Mann J.L., Roessler J.M. (2021). Fumarate is a terminal electron acceptor in the mammalian electron transport chain. Science.

[60] Kumar R., Landry A.P., Guha A., Vitvitsky V., Lee H.J., Seike K. (2022). A redox cycle with complex II prioritizes sulfide quinone oxidoreductase-dependent H_2_S oxidation. J. Biol. Chem..

[61] Ijssennagger N., van der Meer R., van Mil S.W.C. (2016). Sulfide as a mucus barrier-breaker in inflammatory bowel disease?. Trends Mol. Med..

[62] Stummer N., Feichtinger R.G., Weghuber D., Kofler B., Schneider A.M. (2023). Role of hydrogen sulfide in inflammatory bowel disease. Antioxidants (Basel).

[63] Chen B., Lynn-Nguyen T.M., Jadhav P., Halligan B.S., Rossiter N.J., Guerra R.M. (2024). BRD4-mediated epigenetic regulation of endoplasmic reticulum-mitochondria contact sites is governed by the mitochondrial complex III. bioRxiv.

[64] Sun M.G., Williams J., Munoz-Pinedo C., Perkins G.A., Brown J.M., Ellisman M.H. (2007). Correlated three-dimensional light and electron microscopy reveals transformation of mitochondria during apoptosis. Nat. Cell Biol..

[65] Schindelin J., Arganda-Carreras I., Frise E., Kaynig V., Longair M., Pietzsch T. (2012). Fiji: an open-source platform for biological-image analysis. Nat. Methods.

[66] Nowinski S.M., Solmonson A., Rusin S.F., Maschek J.A., Bensard C.L., Fogarty S. (2020). Mitochondrial fatty acid synthesis coordinates oxidative metabolism in mammalian mitochondria. Elife.

[67] Timon-Gomez A., Perez-Perez R., Nyvltova E., Ugalde C., Fontanesi F., Barrientos A. (2020). Protocol for the analysis of yeast and human mitochondrial respiratory chain complexes and supercomplexes by blue native electrophoresis. STAR Protoc..

[68] Jha P., Wang X., Auwerx J. (2016). Analysis of mitochondrial respiratory chain supercomplexes using blue native polyacrylamide gel electrophoresis (BN-PAGE). Curr. Protoc. Mouse Biol..

[69] Cunniff B., McKenzie A.J., Heintz N.H., Howe A.K. (2016). AMPK activity regulates trafficking of mitochondria to the leading edge during cell migration and matrix invasion. Mol. Biol. Cell.

