# Supplementary material for: H2S remodels mitochondrial ultrastructure and destabilizes respiratory supercomplexes
Source: J Biol Chem. 2025 Mar 20;301(5):108433. doi: 10.1016/j.jbc.2025.108433 (PMC12022479; doi:10.1016/j.jbc.2025.108433)
Supplement: Supplementary Information [file mmc1.docx]

H_2_S remodels mitochondrial ultrastructure and destabilizes respiratory supercomplexes

David A. Hanna^1^, Brandon Chen^2,3^, Yatrik M. Shah^2,4^, Oleh Khalimonchuk^5^, Brian Cunniff^6^ and Ruma Banerjee^1^*

Departments of ^1^Biological Chemistry, ^2^Molecular and Integrative Physiology, ^4^Internal Medicine (Division of Gastroenterology), ^3^Cellular and Molecular Biology Program, Michigan Medicine, Ann Arbor, MI USA, Department of Biochemistry, ^5^University of Nebraska-Lincoln, Lincoln, NE USA, ^6^Department of Pathology and Laboratory Medicine, University of Vermont Larner College of Medicine, Burlington, Vermont 05405

*Running title: H_2_S remodels mitochondrial morphology*

*Corresponding Author. email address: [rbanerje@umich.edu](mailto:rbanerje@umich.edu)

**Table of Contents**

**Figure S1.** H_2_S induced mitochondrial swelling is reversible

**Figure S2.** Chronic H_2_S decreases mitochondrial networking in HEK293 cells

**Figure S3.** Chronic H_2_S decreases mitochondrial networking in HT1080 cells

**Figure S4.** Chronic H_2_S decreases mitochondrial networking in SW480 cells

**Figure S5.** TEM analysis of H_2_S effects on mitochondria

**Figure S6.** Chronic H_2_S exposure impairs respiration

**Figure S7.** H_2_S induces S-phase cell cycle arrest

**Figure S8**. H_2_S-dependent decrease mitochondrial networking is OMA1-dependent

**Figure S9.** Structures of the A- and C-respirasome highlighting subunits that are affected by H_2_S

**Figure S1.** **H_2_S induced mitochondrial swelling is reversible**. HT-29 mitochondrial morphology is altered by H_2_S (100 ppm, 24 h) but recovers by 48 h after return to growth in the absence of H_2_S under normal growth conditions. Mitochondria were stained with MitoView. And data are representative of 2 independent experiments. The calibration bar shows that the pixel intensity is within detector’s range, i.e., 0-65,535

**Figure S2.** **Chronic H_2_S decreases mitochondrial networking** **in HEK293 cells**. (**A,B**) Mitochondrial morphology in control (A) versus (B) H_2_S-grown cells (100 ppm, 24 h) altered mitochondrial morphology and decreased mitochondrial networking. Two representative images are shown to illustrate variations in response to H_2_S. The calibration bar shows that the pixel intensity is within detector’s range, i.e., 0-65,535. (**C**) Form factor analysis revealed decreased mitochondrial networking (n >20 images) captured from 2 independent experiments conducted with replicate plates. Two-sample unpaired t test was used for the statistical analysis in C.

**Figure S3.** **Chronic H_2_S decreases mitochondrial networking** **in HT1080 cells**. (**A,B**) Mitochondrial morphology in control (A) versus (B) H_2_S-grown cells (100 ppm, 24 h) altered mitochondrial morphology and decreased mitochondrial networking. Two representative images are shown to illustrate variations in response to H_2_S. The calibration bar shows that the pixel intensity is within detector’s range, i.e., 0-65,535. Two calibration bars were used for images from independent experiments to account for differences in staining intensity. (**C**) Form factor analysis revealed decreased mitochondrial networking (n > 20 images) captured from 2 independent experiments conducted with replicate plates. Two-sample unpaired t test was used for the statistical analysis in C.

**Figure S4.** **Chronic H_2_S decreases mitochondrial networking** **in SW480 cells**. (**A,B**) Mitochondrial morphology in control (A) versus (B) H_2_S-grown cells (100 ppm, 24 h) altered mitochondrial morphology and decreased mitochondrial networking. Two representative images are shown to illustrate variations in response to H_2_S. The calibration bar shows that the pixel intensity is within detector’s range, i.e., 0-65,535. (**C**) Form factor analysis revealed decreased mitochondrial networking (n >20 images) captured from 2 independent experiments conducted with replicate plates. Two-sample unpaired t test was used for the statistical analysis in C.

**Figure S5. Transmission electron microscopy analysis of H_2_S effects on mitochondria. (A,B).** Representative images of ultrastructural changes in mitochondria in control (A) and H_2_S cultured (B, 100 ppm, 24 h) HT-29 cells (scale bar: 1 µM). Ultrastructural morphologies are annotated as described at the top.

**Figure S6. Chronic H_2_S exposure impairs respiration. (A)** Scheme illustrating the experimental setup. The recovery times refer to the duration of culture without sulfide after an initial exposure of HT-29 cells (100 ppm H_2_S, 24 h). (**B,C**) In contrast to control cells, which respond to the addition of 20 µM H_2_S (red arrows) by increasing OCR, H_2_S-grown cells exhibit very low basal OCR and are unresponsive to exogenous sulfide (B,C *top*) but show signs of recovery 24 and 48 h after removal from the H_2_S chamber (B,C, *middle and lower*). (**D, E**) Quantitation of the basal OCR (D) and recovery time (E) data in B and C. The OCR data are representative of 3-4 independent experiments. Two-sample unpaired t test was used for the statistical analyses.

**Figure S7. Sulfide induces S-phase arrest.** (**A,B**) HT-29 were grown ± H_2_S (100 ppm, 24 h) and then allowed to recover for the next 48 h in the absence of sulfide. Flow cytometry analysis of DNA content (B) and quantitation of data (C) indicate S-phase cell-cycle arrest after 24h of sulfide exposure, which is subsequently relieved over 48 h of culture in the absence of H_2_S. The data are representative of 2 independent experiments each conducted in triplicate.

**Figure S8. H_2_S-dependent decrease in mitochondrial networking is OMA1-dependent**. (**A**) Representative microscopy images of wild-type and OMA1 KO MEF cells (cultured ± 100 ppm H_2_S, 24 h). OMA1 KO cells exhibit lower intensity for mitochondrial staining and H_2_S enhances MitoView Green staining in both samples. The calibration bar shows that the pixel intensity is within detector’s range, i.e., 0-65,535. (**B**) Mitochondrial networks were estimated from the relative form factor of mitochondria (n = 19 to 22 images per condition from two independent experiments each conducted in duplicate). In contrast to wild-type cells, mitochondria in OMA1 KOs are less impacted by H_2_S exposure. Two-sample unpaired t test was performed for the statistical analysis in B.

**Figure S9. Structures of the A- and C-respirasome highlighting subunits that are affected by H_2_S. (A, B**) Surface representation of the A- and C-type respirasomes (PDB [8PW5](https://doi.org/10.2210/pdb8PW5/pdb) and [8PW6](https://doi.org/10.2210/pdb8PW6/pdb)) depicting CI (gray), CIII dimer (CIII_2_, yellow) and CIV with COX7A2 (CIV, blue). In the A-respirasome, CIV_c_ binds exclusively to CI and COX7A2 is not predicted to contribute to the stability of the CI and CIV interaction. In the C-respirasome, CIV_c_ binds to CI and CIII_2_ and COX7A2 appears to be necessary for stabilizing CIV_c_ in this supercomplex. The CI and IV subunits that decrease in abundance in response to H_2_S are shown in red. Loss of these protein subunits, especially COX7A2 predict instability and loss of CIV from the respirasome.
